# Quality of life in adults with celiac disease in Spain over a decade

**DOI:** 10.1038/s41598-026-40300-4

**Published:** 2026-03-25

**Authors:** Laura Suárez-Bárcena González, Julián Rodríguez-Almagro, Alberto Bermejo-Cantarero, Antonio Hernández-Martínez, María Laura Parra-Fernandez, Cristina Romero-Blanco

**Affiliations:** 1 Department of Nursing, Ciudad Real School of Nursing, University of Castilla La-Mancha, Ciudad Real, Spain; 2 Instituto de Investigación Sanitaria de Castilla-La Mancha (IDISCAM), Toledo, Spain

**Keywords:** Celiac disease, Quality of life, Gluten-free diet, Population health, Chronic disease management, Diseases, Gastroenterology, Health care, Medical research

## Abstract

Celiac disease (CD) can significantly impair health-related quality of life (HRQOL), mainly due to persistent symptoms and the burden of a lifelong gluten-free diet (GFD). Evidence regarding changes in HRQOL in recent years is scarce despite advances in diagnosis and dietary support. We conducted a comparative cross-sectional study using two large national cross-sectional samples of Spanish adults with CD assessed in 2014 and 2024. HRQOL was measured using the validated Celiac Disease Quality of Life (CD-QOL) questionnaire. Descriptive, bivariate, and multivariable regression analyses were performed to explore changes over time and factors associated with better outcomes. We analyzed 2254 surveys (1208 from 2014; 1046 from 2024). Overall HRQOL remained stable across the decade. Longer duration since diagnosis and more years on a GFD were consistently associated with better emotional and functional well-being. Age showed a modest positive association with HRQOL, while women reported poorer scores in health-concerns domains. Despite increased disease awareness and improvements in gluten-free product availability in Spain over the past decade, perceived quality of life has not meaningfully improved. Quality of life in adults with CD in Spain has not changed over ten years, suggesting that dietary management alone might be insufficient to achieve optimal well-being. Early diagnosis and structured long-term follow-up, including psychological support, could help address persistent emotional and social challenges. Strategies that go beyond diet are needed to enhance patient-centred outcomes in CD.

## Introduction

Celiac disease (CD) is an autoimmune disease that predominantly affects females and can appear at any age^1–3^. It is characterized by an abnormal immune response to gluten in individuals who are genetically susceptible. After ingestion, it can cause intestinal and extraintestinal signs and symptoms. The clinical presentation varies widely between individuals^1–4^.

Currently, the prevalence is estimated to be around 1%, with variations among different regions of the world^1,3,5,6^. However, several authors believe that the true frequency of this disease is underestimated^2^.

Diagnosis is based on the assessment of signs and symptoms suggestive of CD, histopathological evaluation through intestinal biopsy, serological testing, and response to a gluten-free diet (GFD), which is currently the only effective treatment for this disease^1–3^. GFD is currently the standard treatment, as it controls symptoms, promotes villi regrowth, and normalizes antibodies. Furthermore, it reduces the risk of malnutrition, osteoporosis, obstetric or gynecological problems, and cancer^1,3,7^. However, GFD is restrictive, expensive, and carries a risk of cross-contamination during social events and with medications; moreover, symptoms may persist in some patients, which could negatively affect health-related quality of life (HRQoL)^2–4,8–10^. Therefore, appropriate education, motivation, and follow-up are essential.

HRQOL is a key outcome measure that assesses the impact of perceived health on an individual’s life, including physical, psychological, and social functioning^11^. It is a complex construct, influenced by beliefs, perceptions, and life situation^11^. It can be measured with validated instruments. Generic measures, such as the SF-36 Health Survey (SF-36), allow comparisons between diseases and with the general population^12,13^. Specific measures capture aspects specific to each pathology. For CD, the CD-QOL survey stands out, validated by Dorn et al. in 2010^14^ and adapted to Spanish by Casellas et al. in 2013^15^.

As in other chronic diseases, HRQOL is a key outcome in CD. It allows for estimating the population impact and, when combined with other variables, identifying the factors that influence patient well-being^2,16,17^. This information helps assess the effect of interventions and offer more comprehensive care, beyond symptoms and laboratory parameters^2,16,17^. In this regard, in 2014, Rodríguez et al.^16^ conducted a study in Spain with 1230 subjects (89.2% women). They assessed age, sex, time since diagnosis, and duration of GFD and concluded that several of these factors are associated with HRQOL.

However, daily life has changed over the last decade, including for those living with CD. It is important to reassess whether these changes are reflected in HRQOL and the influence of the factors previously described.

The objective of this study was to analyze whether the perception of quality of life in patients with celiac disease has changed in the current context. To this end, two large cross-sectional national samples of Spanish adults with CD (2014 and 2024) were compared. This analysis aimed to assess whether the perception of HRQOL in adults with CD has changed over the last decade in Spain and to determine the factors associated with improved outcomes.

## Methods

### Design and subject selection

This was a cross-sectional study comprising two independent cross-sectional samples, separated by a 10-year interval. A total of 2254 subjects participated: 1208 in the 2014 sample and 1046 in the 2024 sample.

For the 2014 sample, the database of Rodríguez et al.^16^ was used, the results of which were published in 2015.

To obtain data for the 2024 sample, patients were contacted anonymously via a link invitation sent through various associations for people with celiac disease in the country. This invitation included information about the study, a consent form to express their agreement to participate, and an email address to clarify any questions that might arise.

Individuals aged 18 years or older residing in Spain, with a documented medical diagnosis of CD in their medical records, and who had signed the informed consent form prior to participating in the study, were included in the study. Participants who did not meet any of the above criteria or who presented incomplete data on the questionnaire were excluded.

## Variables

The following independent variables were considered: age, sex, time since CD diagnosis, and duration of the GFD. These are the same variables included in the study of the 2014 cross-sectional sample by Rodríguez et al.^16^.

### Health-related quality of Life

The CD-QOL questionnaire, a specific instrument for assessing HRQOL in people with CD, was used as the dependent variable. This questionnaire was validated by Dorn et al. in 2010^14^ and subsequently linguistically and culturally adapted to Spanish by Casellas et al. in 2013^15^.

HRQOL scores were calculated according to the Spanish version of the CD-QOL survey^15^. This questionnaire consists of 20 questions answered on a 5-point Likert scale. The questions are grouped into four dimensions: limitations (ability to enjoy life), dysphoria (frustration, sadness, anger, irritability, and anxiety), health problems (worry about the disease and its complications), and inadequate treatment (management with the GFD). The total score is expressed from 0 to 100 points. Values greater than or equal to 70 points are interpreted as indicating a good HRQOL.

### Statistical analysis

For statistical analysis, qualitative variables were described in terms of absolute frequencies, while quantitative variables were expressed as mean values (mean) and standard deviations (SD).

In bivariate analysis, Pearson’s chi-square test was used for qualitative independent and dependent variables. For quantitative dependent variables, the Student-Fisher t-test and analysis of variance (ANOVA) were applied.

In addition to descriptive and bivariate analyses by cohort, a joint multivariate model was constructed, incorporating both cohorts, to identify independent predictors of quality of life and estimate the net effect of the study year, while adjusting for potential confounders. In these analyses, the dependent variable was the overall score of the quality of life index, along with its four dimensions, while the independent variables included year of study, sex, patient age, time to diagnosis, and duration of treatment. Mean differences (MD) and adjusted mean differences (aMD) were estimated with their respective 95% confidence intervals.

Statistical analysis was performed using SPSS version 29.0.

## Ethical and legal considerations

This study used a convenience sample of individuals with CD from across the country, aged 18 years and older, who agreed to participate in the study. This study was approved by the Social Research Ethics Committee (CEIS, *for its initials in Spanish*) of the University of Castilla La Mancha (Registration No.: CEIS-2024-36901), and was conducted in accordance with the guidelines of the 1964 Declaration of Helsinki and its subsequent revisions.

## Results

A total of 2254 questionnaires were included in the analysis, comprising 1208 from 2014 to 1046 from 2024. The majority were women in both samples (89.2% and 87.8%, respectively), with a mean age of 33.3 years (SD: 10.12). Table 1 shows the information for the two cross-sectional samples, differentiated by gender, age, time since CD diagnosis, and duration of GFD follow-up. The reported *p* values correspond to within-year sex comparisons for each variable and do not assess differences between the 2014 and 2024 samples. Differences between study years were evaluated separately using multivariable models, in which study year was included as an independent predictor (Table 3).

**Table 1:** Characteristics of the two cross-sectional samples, according to gender, age, time since diagnosis of CD, and years of GFD.

		Year 2014				Year 2024			
		Total 1208	Males 130	Females 1078	Value *p*	Total 1046	Males 127	Females 919	Value *p*
Age (years)									
Mean (SD)		33.3 ± 10.12	33.6 ± 9.40	33.2 ± 10.20	0.666	36.4 ± 11.26*	37.5 ± 12.55*	36.3 ± 11.06*	0.267*

Category		N (%)	N (%)	N (%)		N (%)	N (%)	N (%)	
Up to 20 years		84 (7.0)	9 (6.9)	75 (7.0)	0.222	48 (4.6)	9 (7.1)	39 (4.2)	0.257
21–30 years		450 (37.3)	41 (31.5)	409 (37.9)		330 (31.5)	33 (26.0)	297 (32.3)	
31–40 years		410 (33.9)	55 (42.3)	355 (32.9)		297 (28.4)	31 (24.4)	266 (28.9)	
41–50 years		186 (15.4)	21 (16.2)	165 (15.3)		248 (23.7)	35 (27.6)	213 (23.2)	
51–60 years		64 (5.3)	3 (2.3)	61 (5.7)		101 (9.7)	15 (11.8)	86 (9.4)	
> 60 years		14 (1.2)	1 (0.8)	13 (1.2)		22 (2.1)	4 (3.1)	18 (2)	
Time diagnosed (years)									
Mean (SD)		9.2 ± 9.44	10.0 ± 9.78	9.1 ± 9.39	0.311	10.2 ± 9.50*	9.8 ± 9.09*	10.3 ± 9.55*	0.570*
Up to 1 year		211 (17.5)	14 (10.8)	197 (18.3)	0.322	123 (11.8)**	12 (9.5)**	111 (12.1)**	0.849*
> 1 year − 5 years		413 (34.2)	49 (37.7)	364 (33.8)		301 (28.9)**	39 (31.0)**	262 (28.6)**	
6–10 years		201 (16.6)	22 (16.9)	179 (16.6)		245 (23.5)**	32 (25.4)**	213 (23.2)**	
11–15 years		114 (9.4)	13 (10.0)	101 (9.4)		111 (10.6)**	14 (11.1)**	97 (10.6)**	
> 15 years		269 (22.3)	32 (24.6)	237 (22.0)		263 (25.2)**	29 (23.0)**	234 (25.5)**	
Years on GFD									
Mean (SD)		8.8 ± 9.14	10.1 ± 9.88	8.7 ± 9.04	0.091	10.0 ± 9.15*	9.7 ± 9.12*	10.1 ± 9.16*	0.685*
Up to 1 year		223 (18.5)	14 (10.8)	209 (19.4)	0.200	125 (12.0)**	13 (10.2)**	112 (12.2)**	0.892*
> 1 year − 5 years		418 (34.6)	48 (36.9)	370 (34.3)		301 (28.9)**	38 (29.9)**	263 (28.7)**	
6–10 years		197 (16.3)	23 (17.7)	174 (16.1)		249 (23.9)**	33 (26.0)**	216 (23.6)**	
11–15 years		116 (9.6)	13 (10.0)	103 (9.6)		114 (10.9)**	15 (11.8)**	99 (10.8)**	
> 15 years		254 (21.0)	32 (24.6)	222 (20.6)		254 (24.4)**	28 (22.0)**	226 (24.7)**	

*Missing 6 (N: 1040)

**Missing 3 (N: 1043)

In 2014, the mean age was 33.3 years (SD: 10.12); the mean time since diagnosis was 9.2 years (SD: 9.44), and the time on the gluten-free diet was 8.8 years (SD: 9.14). Regarding the 2024 sample, the mean age was 36.4 years (SD: 11.26); the mean time since diagnosis was 10.2 years (SD: 9.50), and the time on the gluten-free diet was 10.0 years (SD: 9.15). No statistically significant differences were found between groups (*p* > 0.05).

### Evolution of HRQOL between 2014 and 2024

**Fig. 1:**
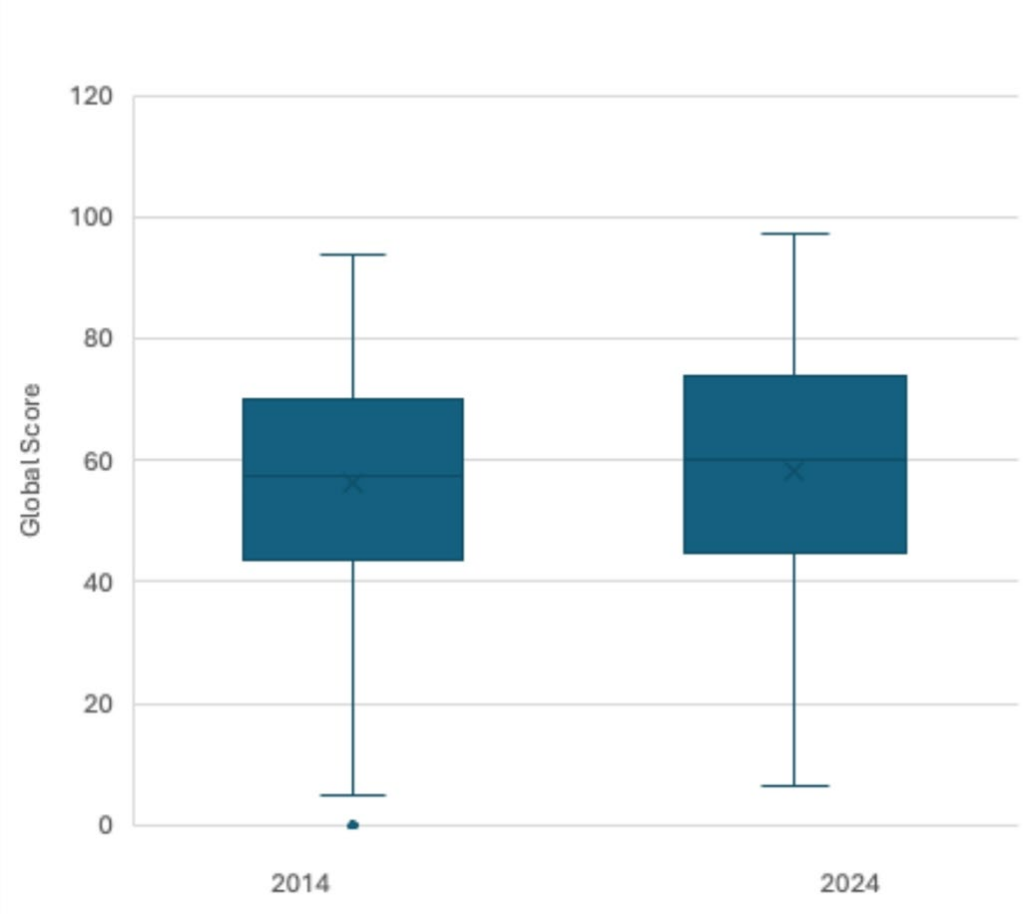
Comparison of the mean total score obtained on the CD-QOL between 2014 and 2024 cohorts.

The overall CD-QOL scores were similar: 56.47 ± 18.10 in 2014 and 58.13 ± 18.94 in 2024 (Fig. 1). In both samples, the highest-scoring dimension was “dysphoria,” and the lowest, “inadequate treatment.” No significant differences were detected in the multivariate analysis between the study years (adjusted MD = 0.67; 95% CI −0.81 to 2.15; *p* = 0.375).

Table 2 shows the overall and dimension scores of the CD-QOL questionnaire according to gender, age, time since diagnosis, and years on GFD.

**Table 2:** Global and dimensional analysis of the CD-QOL questionnaire for each cross-sectional sample, broken down by gender, age, time since diagnosis, and years on GFD.

	Year 2014					Year 2024				
	Global score M (SD)	Dysphoria M (SD)	Limitations M (SD)	Health problems M (SD)	Inadequate treatment M(SD)	Global score M (SD)	Dysphoria M (SD)	Limitations M (SD)	Health problems M (SD)	Inadequate treatment M(SD)
Gender										
Man	58.72 ± 17.90	84.37 ± 16.36	54.08 ± 23.16	57.69 ± 25.59	30.86 ± 19.13	59.61 ± 19.96	81.54 ± 19.14	54.76 ± 24.66	60.39 ± 28.69	35.62 ± 20.11
Women	56.20 ± 18.11	81.23 ± 19.68	52.43 ± 23.25	50.72 ± 25.85	36.75 ± 20.95	57.93 ± 18.80	81.00 ± 20.50	52.42 ± 24.07	58.25 ± 25.53	35.74 ± 20.79
*P* value	0.134	**0.045**	0.446	**0.004**	**0.002**	0.348	0.779	0.306	0.427	0.953
Age										
Upto 20 years	62.51 ± 18.37	86.53 ± 14.60	57.24 ± 23.58	63.27 ± 28.19	36.30 ± 20.22	59.97 ± 16.18	83.98 ± 17.53	51.33 ± 21.27	65.93 ± 22.13	35.93 ± 20.56
21–30 years	56.40 ± 17.49	81.77 ± 18.07	51.88 ± 22.57	52.20 ± 26.45	36.55 ± 20.65	56.30 ± 19.54	79.05 ± 21.47	50.08 ± 24.49	57.46 ± 26.08	35.87 ± 20.94
31–40 years	54.85 ± 18.05	81.18 ± 20.34	51.30 ± 23.60	48.01 ± 24.81	35.24 ± 19.76	55.22 ± 18.32	79.10 ± 20.99	49.16 ± 23.53	54.46 ± 24.85	36.61 ± 20.49
41–50 years	56.63 ± 17.65	80.94 ± 19.93	53.73 ± 22.05	50.96 ± 23.80	35.21 ± 22.54	59.73 ± 18.03	83.54 ± 17.92	55.36 ± 23.78	58.87 ± 25.59	33.97 ± 20.03
51–60 years	57.50 ± 21.32	77.44 ± 24.66	54.73 ± 26.55	52.96 ± 27.07	41.40 ± 24.38	64.22 ± 20.18	83.72 ± 21.48	61.57 ± 23.60	64.65 ± 28.42	36.01 ± 22.31
> 60 years	62.76 ± 21.04	83.48 ± 18.11	61.90 ± 27.55	58.92 ± 26.75	34.82 ± 19.72	75.00 ± 10.91	91.47 ± 10.66	72.34 ± 18.13	80.68 ± 19.35	39.77 ± 21.00
*P* value	**0.012**	0.111	0.142	**< 0.001**	0.364	**< 0.001**	**0.003**	**< 0.001**	**< 0.001**	0.668
*P* value linear trend	0.815	0.326	0.325	0.669	0.821	**< 0.001**	**0.038**	**< 0.001**	**0.004**	0.540
Time diagnosed (years)										
Up to 1 year	51.93 ± 18.74	72.92 ± 22.55	46.45 ± 23.08	49.78 ± 25.85	39.92 ± 19.42	48.98 ± 19.24	65.34 ± 24.24	44.91 ± 23.97	46.99 ± 25.22	39.53 ± 21.55
> 1 year − 5 years	52.65 ± 17.22	77.99 ± 20.09	48.11 ± 22.21	48.03 ± 24.22	33.95 ± 21.52	52.10 ± 19.20	74.79 ± 22.14	45.30 ± 23.71	52.24 ± 25.67	37.04 ± 20.72
6–10 years	56.69 ± 16.29	84.39 ± 16.63	51.64 ± 21.80	51.89 ± 24.51	36.00 ± 19.82	58.92 ± 17.24	84.31 ± 16.00	52.33 ± 22.65	59.71 ± 25.10	35.86 ± 20.33
11–15 years	61.96 ± 17.21	88.26 ± 15.95	60.52 ± 22.21	55.52 ± 26.93	31.90 ± 20.71	62.72 ± 16.44	86.88 ± 15.92	55.38 ± 22.43	68.60 ± 22.75	32.77 ± 20.22
> 15 years	63.39 ± 17.92	88.89 ± 14.15	61.71 ± 22.84	56.05 ± 28.18	38.33 ± 21.03	66.52 ± 16.80	89.97 ± 14.20	63.87 ± 22.30	65.70 ± 24.91	33.60 ± 20.43
*P* value	**< 0.001**	**< 0.001**	**< 0.001**	**< 0.001**	**< 0.001**	**< 0.001**	**< 0.001**	**< 0.001**	**< 0.001**	**0.033**
*P* value linear trend	**<0.001**	**<0.001**	**<0.001**	**< 0.001**	0.234	**< 0.001**	**< 0.001**	**< 0.001**	**< 0.001**	**0.001**
Years on GFD										
Up to 1 year	51.17 ± 18.77	71.74 ± 23.02	46.18 ± 23.16	48.20 ± 25.73	39.85 ± 19.27	49.88 ± 19.11	66.35 ± 24.30	46.31 ± 23.87	47.24 ± 25.44	39.60 ± 21.63
> 1 year − 5 years	52.96 ± 16.90	78.52 ± 19.44	48.13 ± 21.77	48.46 ± 24.13	34.80 ± 21.93	51.81 ± 19.26	74.77 ± 21.93	44.76 ± 23.87	52.19 ± 25.39	36.66 ± 20.74
6–10 years	56.90 ± 16.72	84.51 ± 16.74	51.88 ± 22.14	52.28 ± 24.84	35.85 ± 19.77	59.04 ± 17.08	84.26 ± 16.00	52.74 ± 22.46	59.39 ± 25.36	36.04 ± 20.26
11–15 years	61.91 ± 17.14	88.25 ± 15.72	60.44 ± 21.89	55.77 ± 26.84	31.25 ± 19.67	62.67 ± 16.66	86.18 ± 17.31	55.38 ± 22.43	68.55 ± 22.55	33.77 ± 21.03
> 15 years	64.06 ± 17.77	89.86 ± 13.28	62.60 ± 22.96	56.71 ± 28.19	37.45 ± 21.10	66.87 ± 16.83	90.55 ± 13.72	64.14 ± 22.37	66.37 ± 24.80	33.12 ± 20.24
*P* value	**< 0.001**	**< 0.001**	**< 0.001**	**< 0.001**	**0.003**	**< 0.001**	**< 0.001**	**< 0.001**	**< 0.001**	**0.039**
*P* value linear trend	**< 0.001**	**< 0.001**	**< 0.001**	**< 0.001**	0.057	**< 0.001**	**< 0.001**	**< 0.001**	**< 0.001**	**0.002**

*SD* Standard deviation.

Significant values are in bold at *p* < 0.05.

Regarding gender, in 2014, women scored lower than men on the “dysphoria” dimension (81.23 vs. 84.37; *p* = 0.045) and “health problems” (50.72 vs. 57.69; *p* = 0.004), but higher on “inappropriate treatment” (36.75 vs. 30.86; *p* = 0.002). No differences were found for the “limitations caused by the disease” dimension. In 2024, no significant differences were observed in either the overall score or any of its dimensions.

Regarding age, in both samples, those over 60 years of age obtained the highest scores overall and by dimension. In 2024, scores progressively improved with age, being statistically significant for all dimensions except “inappropriate treatment.” The older the patient, the better the adaptation and perception of quality of life, except for access to and quality of treatment, which remained low in all groups.

Both the time since diagnosis and the number of years on the GFD showed a positive association with HRQOL. Patients with a longer history of diagnosis or more years on the diet scored better on most dimensions. However, the “inadequate treatment” dimension remained the lowest-scoring, without showing improvement over time; in fact, in 2024, it showed an inverse linear trend.

In the multivariate analysis (Table 3), the year of the study (2014 vs. 2024) was not significantly associated with the overall CD-QOL score (adjusted MD = 0.67; 95% CI −0.81 to 2.15; *p* = 0.375). No significant differences were observed by year in any of the dimensions, except for “health problems,” where 2024 was associated with higher scores (MD = 6.44; 95% CI 4.29 to 8.59; *p* < 0.001).

**Table 3:** Multivariate analysis using multiple linear regression between quality of life and its dimensions in terms of year of study, sex, age, time since diagnosis and years on GFD.

	Global score		Dysphoria		Limitations		Health problems		Inadequate treatment	
	MD (95% CI)	*P* value	MD (95% CI)	*P* value	MD (95% CI)	*P* value	MD (95% CI)	*P* value	MD (95% CI)	*P* value
Year	0.67 (−0.81;2.15)	0.375	−1.39 (−2.97;0.19)	0.085	−1.40 (−3.30;0.48)	0.146	**6.44 (4.29;8.59)**	**< 0.001**	−0.27 (−2.01;1.46)	0.758
Sex	1.67 (−0.63;3.97)	0.155	1.44 (−1.01;3.89)	0.249	1.39 (−1.54;4.33)	0.353	**4.27 (0.94;7.60)**	**0.012**	**−3.11 (−5.82;−0.41)**	**0.024**
Age	**0.09 (0.02;0.15)**	**0.011**	0.02 (−0.04;0.09)	0.508	**0.18 (0.09;0.27)**	**< 0.001**	0.01 (−0.09;0.10)	0.875	−0.01 (−0.09;0.06)	0.733
Time diagnosed	**0.42 (0.21;0.62)**	**< 0.001**	**0.58 (0.36;0.80)**	**< 0.001**	**0.51 (0.25;0.78)**	**< 0.001**	**0.39 (0.09;0.69)**	**0.009**	**−0.26 (−0.50;−0.02)**	**0.032**
Years on GFD	0.17 (−0.02;0.37)	0.082	0.09 (−0.11;0.30)	0.375	**0.24 (−0.01;0.50)**	**0.054**	0.08 (−0.20;0.37)	0.575	**0.24 (0.01;0.47)**	**0.041**

*MD* Mean difference;* CI* Confidence Interval.

Significant values are in bold.

Regarding gender, being female was associated with a significantly lower score on the “health problems” dimension (MD = 4.27; 95% CI 0.94 to 7.60; *p* = 0.012) and with a higher score on “inadequate treatment” (MD = − 3.11; 95% CI −0.81 to −0.41; *p* = 0.024).

Age showed a positive and significant association with the overall CD-QOL score (MD per year = 0.09; 95% CI 0.02 to 0.15; *p* = 0.011), as well as with the “limitations caused by the disease” dimension (MD = 0.18; 95% CI 0.09 to 0.27; *p* < 0.001).

Time since diagnosis showed the strongest association with overall quality of life (MD = 0.42 per year; 95% CI 0.21 to 0.62; *p* < 0.001), as well as with the “dysphoria” (MD = 0.58), “limitations caused by the disease” (MD = 0.51), and “health problems” (MD = 0.39) dimensions, all with *p* < 0.01. The duration of the GFD was significantly associated with improvements in “inadequate treatment” (MD = 0.24; 95% CI 0.01 to 0.47; *p* = 0.041), but not in the overall score.

## Discussion

The objective of this study was to analyze how the impact of CD has evolved over the last decade. The study was conducted in Spain with a large national cross-sectional sample by Rodríguez et al.^16^ had already pointed out how the variables age, sex, time since diagnosis, and duration of GFD affect the quality of life of affected individuals. However, the context has changed in the last decade: diagnosis and awareness have improved, and the offer of gluten-free products in supermarkets and restaurants has expanded^18^. Therefore, the study was re-evaluated ten years later using the same questionnaire and comparable methods.

The results showed that, despite social and healthcare changes in the national context, HRQOL remained stable between 2014 and 2024, with moderate overall scores in both cross-sectional samples, and no significant differences were observed after multivariate adjustment. However, factors consistently associated with better perceptions of well-being were identified: time since diagnosis was the strongest predictor of a higher overall score and improvement in the emotional, functional, and physical dimensions of the CD-QOL.

Likewise, age showed a positive effect on the overall score and the “limitations” dimension. In contrast, the “inadequate treatment” dimension remained the lowest-scoring, with no evidence of improvement over time, highlighting the need to advance therapeutic strategies beyond the GFD.

The two samples had comparable sizes and a homogeneous distribution of the variables analyzed. In both, females predominated. This pattern is consistent with other similar studies, using either the same or different HRQOL questionnaires^19–30^. This could be due to a greater willingness of women to participate in associations and studies of this nature^20,21^. Therefore, it is advisable to promote recruitment strategies targeting men to improve representativeness. Even so, the gender distribution of this study is not considered a factor influencing the results, as it was controlled for in the multivariate analysis.

The mean total scores in 2014 (56.47 ± 18.10) and 2024 (58.13 ± 18.94) reflect a moderate HRQOL, with no significant changes over the decade. Notably, the absence of improvement in overall HRQOL over the ten-year period was observed in a sample of adults with celiac disease who are likely to have relatively favorable conditions, including confirmed diagnosis and exposure to disease-related information and support. This suggests that increased awareness and availability of gluten-free products alone may be insufficient to achieve meaningful gains in long-term quality of life. However, comparison with other studies reveals differences attributable to the population profile and methodology. This more heterogeneous approach, reflecting the experience of diagnosed individuals with varying levels of engagement with disease-related care, may translate into relatively lower CD-QOL scores compared to series based on associations or with restrictive criteria^20,22^. This distinction is relevant, as samples drawn exclusively from associations or specialized units typically represent patients with higher levels of engagement and support, which is consistently associated with better HRQOL scores. In line with this, samples from associations or specialized units, with at least one year of GFD and exclusion of comorbidities, tend to score higher^19,21,23,29^. Similarly, other HRQOL instruments confirm this pattern: specific counseling^31^, membership in associations^32^, and a longer duration of GFD^27,31–33^ are associated with better scores. These findings underscore the importance of accessible education and support programs.

This may be explained by several hypotheses. Lower HRQOL may be related to comorbidities and complicated CD or persistent symptoms^25,28,34^. Taken together, these findings highlight the potencial need for a more comprehensive approach. Structured follow-up and support promote sustained adherence to the GFD and improve quality of life^24,35^. Along these lines, at the Columbia Cancer Center^24^, greater satisfaction with clinical time was associated with better CD-QOL scores (75.90 vs. 69.18; *p* = 0.002). Zarkadas et al.^35^ show that information and support strengthen adherence. In turn, high adherence to the GFD is a priority because it is associated with better HRQOL^19,23,26,29,30,36–40^. Therefore, these findings support the inclusion of adherence programs with ongoing monitoring and support as part of standard care. This ongoing support strengthens the professional-patient relationship, facilitates the early identification of barriers, and provides personalized strategies that boost motivation.

Regarding gender, in 2024, no significant differences in HRQOL were observed. This finding is consistent with recent studies using the CD-QOL and other instruments, which show a less marked gap than a decade ago^19,20,22,23,28–30,32^. However, we also find current studies that report significant differences^27,36,37^. The results are disparate and therefore inconclusive. Studies with greater male representation and the application of sex stratification are needed to identify differential personal factors and plan gender-centered care.

A finding that is repeated ten years later, and in line with other studies, is that older patients have better HRQOL scores than younger patients^20–23^. This finding is compatible with the hypothesis of Castilhos et al.^21^: greater experience with GFD and most common household habits would explain the better HRQOL. Therefore, it is necessary to promote new treatments, expand the availability of gluten-free products in supermarkets and restaurants, and strengthen health education and support for young people.

Regarding the results obtained in the different dimensions, “inappropriate treatment” had the lowest scores, in line with other studies^16,38^. The persistently low score is consistent with the current context: there are no alternatives to GFD, and its implementation requires strict control and trace monitoring. Therefore, finding new alternatives to GFD treatment is a priority, and as noted by Lee et al.^20^, food goes beyond nutrition and functions as a space for social interaction. Likewise, the public health framework (labeling, traceability, safe supply) must be strengthened^18^. In the remainder, “dysphoria” was the highest-scoring; “limitations caused by the disease” remained in the intermediate range; and “health problems” showed improvement in 2024. All improved with time from diagnosis and with years on the GFD, which is supported by early emotional support, practical education for social life, and prevention of cross-contamination, and periodic clinical follow-up.

Finally, it is worth noting that overall HRQOL scores increased with time from diagnosis and from the start of the GFD. This pattern is consistent in the literature, regardless of the instrument^20–22,26,31–33,37^. Furthermore, good adherence to the GFD has been shown to be a determining factor in the perception of well-being and in the reduction of complications associated with CD^19,23,26,29,30,36–40^. In our sample, time since diagnosis was the factor with the greatest impact, also in several dimensions. The findings support early diagnosis and ongoing follow-up aimed at sustained adherence to the GFD. This mitigates the impact of restrictions and promotes adaptation.

A strength of our study is that it is the first to evaluate the ten-year comparison of HRQOL in adults with CD in Spain, using two large national cross-sectional samples. The methodological replication and the homogeneity of the samples (sex, age, time since diagnosis, and duration of the GFD; comparable means and SD) reinforce the robustness and comparability of the findings across time.

Several limitations of this study should be acknowledged. First, the cross-sectional design precludes causal inference regarding changes in health-related quality of life over time. Second, participants were recruited through celiac disease associations, which may introduce selection or participation bias. Individuals affiliated with patient organizations are likely to be more informed, more engaged in disease management, and to have greater access to educational and social support resources than non-affiliated patients; therefore, the findings may not be fully generalizable to the entire population of adults with celiac disease in Spain. Third, male participation was low, which may have limited the ability to detect small sex-related differences. Finally, several relevant determinants of health-related quality of life were not assessed, including adherence to the gluten-free diet, persistence of symptoms, comorbidities, and socioeconomic or educational factors. These variables may play an important role in shaping patient-reported outcomes and should be incorporated into future population-based studies.

## Conclusions

In this study, the overall mean CD-QOL score was 56.47 in 2014 and 58.13 in 2024, placing the results at a moderate level and leaving room for improvement of ~ 12 points to the threshold for “good” HRQOL.

In 2014, better values ​​were observed in older individuals and those with longer duration on the GFD. Currently, these variables remain decisive for well-being and quality of life.

The findings suggest that a better quality of life in people with CD is associated with adequate clinical counseling and follow-up, greater adherence to and duration of the GFD, advancing age, and the absence of persistent symptoms or comorbidities.

Therefore, these findings support the prioritization of strategies that sustainably improve these factors. In particular, guidelines aimed at early diagnosis, allowing for initiation of the GFD as early as possible, and strategies that promote high adherence through adequate follow-up should be prioritized. Furthermore, it is crucial to analyze the needs of the younger population and people with complicated CD or comorbidities in order to develop targeted interventions that improve their quality of life.

## Data Availability

The datasets used and analyzed during the current study are available from the corresponding author on reasonable request.
